# Editorial: Novel techniques of minimally invasive spine surgery for various pathologies

**DOI:** 10.3389/fsurg.2023.1267438

**Published:** 2023-09-01

**Authors:** Yutong Gu, Qingquan Kong, William Lavelle

**Affiliations:** ^1^ Department of Orthopaedic Surgery, Zhongshan Hospital, Fudan University, Shanghai, China; ^2^ Shanghai Southwest Spine Surgery Center, Shanghai, China; ^3^ Department of Orthopaedic Surgery, West China Hospital of Sichuan University, Sichuan, China; ^4^ SUNY Upstate Medical University, Syracuse, NY, United States

**Keywords:** minimally invasive spine surgery, spinal endoscopic surgery, PVP and PKP, MIS-TLIF, OLIF, mini-open or channel assisted surgery, navigation-assisted spine surgery, hybrid minimally invasive surgical techniques

**Editorial on the Research Topic** Novel techniques of minimally invasive spine surgery for various pathologies

In recent years, minimally invasive spine surgery (MISS) has been rapidly developed as its concepts and techniques keep evolving and spreading. This Research Topic focuses on novel techniques related to MISS and covers almost all aspects of MISS treatment of spine trauma, degeneration, tumor, and infection.

## 1. Spinal endoscopic surgery

Spinal endoscopic surgery is a rapidly developing technique over the past 30 years and has been widely used in the treatment of spinal disorders including degenerative diseases with neurological symptoms.

### 1.1. Percutaneous transforaminal endoscopic surgery (PTES)

Yeung endoscopic spine system (YESS) (1) and transforaminal endoscopic spine system (TESS) (2) are two of the most classic techniques. YESS is a type of “in-out” technique. This technique involves first entering the intervertebral disc and gradually incising the tissue from the inside out. TESS is a type of “out-in” technique. This technique involves entering the spinal canal through the foramina and removing the herniated nucleus pulposus from the outside in. The two techniques are still widely used nowadays and act as the basis of many modified transforaminal endoscopic surgeries such as PTES. PTES is a novel, minimally invasive technique that has been used in the treatment of lumbar degenerative diseases (LDDs). Its advantages include simple orientation, facile puncture, reduced number of steps, minimal x-ray exposure, and shortened operation time.

Zhou et al. used PTES under local anesthesia to treat the culprit segment of LDDs predicted by radiologic images (Group A) or clinical symptoms (Group B). Group B showed a significantly lower operative duration, lower blood loss, and a lower fluoroscopy frequency than group A (*P* < 0.001). The VAS score of leg pain and the ODI score significantly dropped after operation in both groups (*P* < 0.001), and the excellent and 28 good rate was 97.6% (41/42) in group A and 100% (45/45) in group B at the 2-year follow-up. The results showed that it is much more accurate to predict the culprit segment according to clinical symptoms than using radiologic images.

Zhou et al. compared PTES for surgical treatment of LDD in elderly patients with minimally invasive surgery-transforaminal lumbar interbody fusion (MIS-TLIF). The authors found that the PTES group showed a significantly lower operation time (55.6 ± 9.7 min vs. 97.2 ± 14.3 min, *P* < 0.001), lower blood loss [11 (2–32) ml vs. 70 (35–300) ml, *P* < 0.001], shorter incision length (8.4 ± 1.4 mm vs. 40.6 ± 2.7 mm, *P* < 0.001), lower fluoroscopy frequency times [5 (5–10) vs. 7 (6–11), *P* < 0.001], and shorter hospital stays [3 (2–4) days vs. 7 (5–18) days, *P* < 0.001] than the MIS-TLIF group. PTES was also performed under local anesthesia. The PTES technique should be preferred when patients have no spinal instability.

Lisheng et al. described one case of central calcified thoracic disc herniation (CCTDH) treated with modified PTES via a unilateral posterolateral approach under local anesthesia and conscious sedation, with the help of a flexible power diamond drill. The authors drew the conclusion that modified PTES may be an alternative, minimally invasive technique for the treatment of CCTDH and may provide similar or better outcomes than traditional open surgery.

### 1.2. Unilateral biportal endoscopy (UBE)

The UBE technique places two working cannulas unilaterally, one channel for observation and the other for operation. It has the advantages of broad vision and flexible operation.

Hu et al. investigated the clinical efficacy and imaging outcomes of UBE with unilateral laminotomy for bilateral decompression (ULBD) for the treatment of severe lumbar spinal stenosis (LSS). The mean hospital stay was 2.76 ± 1.02 days. At the final follow-up, the VAS score for back pain and leg pain decreased from 7.22 ± 0.95 to 1.26 ± 0.44 and from 7.88 ± 0.69 to 1.18 ± 0.39, respectively, and the ODI score decreased from 69.88 ± 6.32% to 14.96 ± 2.75%. According to the modified Macnab criteria, the results were excellent in 24 (48%), good in 22 (44%), and fair in 4 (8%). Excellent or good results (a satisfactory outcome) were obtained in 92% of the patients. UBE-ULBD has a good clinical effect in the treatment of severe LSS, and has achieved satisfactory results in spinal canal enlargement, undercutting of facet joints, and decompression effects.

Wang et al. used the UBE technique for the decompression and removal of an extradural mass in five patients. This technique has advantages such as minimizing trauma to normal structures, a magnified endoscopic view, and early recovery after surgery. Biportal endoscopy may be used as an alternative surgical treatment for symptomatic intraspinal extradural benign lesions.

### 1.3. Other spinal endoscopic surgeries

Wang et al. designed a power-aided reciprocating burr for transforaminal endoscopic lumbar discectomy (TELD) and reported the technical details. The results showed that the current clinical data demonstrated the safety and efficacy of modified TELD using a power-aided reciprocating burr for treating lumbar disc herniation (LDH), and this technique significantly reduces the learning curve for beginners when performing foraminoplasty.

Li et al. evaluated the clinical efficacy and safety of percutaneous endoscopic lumbar discectomy (PELD) for the treatment of LDH linked with posterior ring apophysis separation (PRAS). The mean operation time was 118.04 ± 19.31 min and the mean blood loss was 22.84 ± 15.89 ml. The VAS and ODI scores continued to improve from immediately after the surgery to the last follow-up, demonstrating that PELD has reliable efficacy and safety in the treatment of LDH linked with PRAS.

Wei et al. presented an uncommon intraspinal gas-containing synovial cyst treated by percutaneous transforaminal endoscopic cystectomy. A 52-year-old man presented with radicular pain and intermittent claudication that had persisted for 1 month. Computed tomography revealed an intraspinal cystic lesion anteromedial to the left L4/5 articular joint, and the center of the lesion manifested gas contents. A transforaminal endoscopic procedure was performed and was confirmed to be a safe and minimally invasive technique for gas-containing lumbar synovial cysts. It provides a valuable substitution to and supplementation for open surgery.

## 2. Percutaneous vertebroplasty (PVP) and percutaneous kyphoplasty (PKP)

PVP and PKP are surgical methods for the treatment of spinal compression fractures. Bone cement or graft bone are used to restore the interbody height and strengthen the vertebral body. In PKP, balloon dilatation is performed after the puncture needle enters the vertebral body.

Zhou et al. retrospectively analyzed 160 elderly patients of osteoporotic vertebral compression fractures (OVCF) who underwent PVP treatment. The VAS and ODI scores for types I, II, and III were lower 1 year postoperatively than those for types IV and V (*P* < 0.05). One year after surgery, the Cobb angle and the anterior vertebral height ratio of types IV and V were significantly different from those of types I, II, and III (*P* < 0.05), and there was a statistically significant difference between types IV and V (*P* < 0.05). In terms of the incidence of injured vertebral refractures and adjacent vertebral fractures, the evenly distributed types I, II, and III were significantly lower than the unevenly distributed types IV and V, and the incidence of type V was higher (*P* < 0.05). The results showed that the clinical efficacy of cement distribution following PVP of types I, II, and III is better than that of types IV and V, which can better relieve pain, with long-lasting efficacy, and can minimize the occurrence of refractures of injured vertebrae and adjacent vertebral body fractures.

Dai et al. investigated the clinical efficacy and long-term stability of bone cement in the bilateral pedicle anchoring technique with PVP for the treatment of Kümmell disease. The results showed that the bilateral pedicle anchoring technique with PVP integrates the bone cement in the vertebral body and in the pedicle, enhances the stability of the bone cement, and effectively prevents the displacement of the intravertebral bone cement. The postoperative bone cement stability was high, the clinical effect was obvious, and long-term follow-up results were satisfactory. It is a safe and effective surgical method for the treatment of Kümmell disease.

Pusceddu et al. retrospectively evaluated the feasibility and effectiveness of vertebroplasty using spinejack implantation for the treatment and stabilization of painful vertebral compression fractures in patients diagnosed with multiple myeloma (MM), allowing both effective pain reduction and global structural spine stabilization. In the 6-month follow-up, the mean VAS score decreased from 5.4 ± 1.0 to 0.2 ± 0.5, with a mean reduction of 96.3%. The Functional Mobility Scale decreased from 2.3 ± 0.5 to 1.2 ± 0.4, with a mean reduction of −47.8%. These results suggest that vertebroplasty using spinejack implantation for the treatment and stabilization of painful vertebral compression fractures, secondary to MM, is a safe and effective procedure, with long-term pain relief and restoration of vertebral height achieved.

Jiang et al. performed a prospective cohort study to compare the clinical outcomes and radiological parameters of patients undergoing PVP versus those undergoing percutaneous vertebral-disc plasty (PVDP) for back pain, segmental instability, and kyphosis due to very severe thoracolumbar OVCFs. At the last follow-up, the average VAS, ODI, and LKA scores for patients in the PVP group were observed to be higher than those in the PVDP group (*P* < 0.05), which showed that PVDP may be a feasible and effective technique for the treatment of very severe OVCFs, and that it can restore intervertebral height, provide segmental stabilization, and relieve back pain in the short term.

Hao et al. reported a case of shock after PVP for treating OVCF of the fifth thoracic vertebra. An 80-year-old female patient developed shock 90 min after PVP, which was induced by subcutaneous hemorrhage up to 1,500 ml at the puncture site. Before using vascular embolization, transfusion and blood transfusion were used to maintain blood pressure, and local ice bag compression was used to reduce swelling and stop bleeding, which achieved successful hemostasis. The patient recovered and was discharged after 15 days, with the hematoma having absorbed. There was no recurrence during the 17-month follow-up. Although PVP is considered to be a safe and effective method to treat OVCF, surgeons should be vigilant for possible hemorrhagic shock.

Jiang et al. described and evaluated a modified trajectory of PKP for the treatment of OVCF. Eighty-one patients who underwent PKP for lumbar OVCF were divided into an observation group (via the superior pedicle approach) and a control group (via the transpedicular approach). The conclusion was that, compared with the bilateral pedicular approach to PKP for lumbar OVCF, unilateral puncture via the superior pedicle notch can reach the center of the vertebral body to achieve bilateral cement dispersion, reducing the operative time and intraoperative radiation exposure and decreasing the rate of paravertebral cement leakage, while obtaining the same vertebral body height, recovery rate, and clinical efficacy as the bilateral pedicular approach to PKP.

Yu et al. identified risk factors for residual low back pain (LBP) after PKP and developed a nomogram to predict the occurrence of residual LBP. Univariate and multifactorial logistic regression analyses identified depression (*P* = 0.02), intravertebral vacuum cleft (*P* = 0.01), no anti-osteoporosis treatment (*P* < 0.001), cement volume <3 ml (*P* = 0.02), and cement distribution (*P* = 0.01) as independent risk factors for residual LBP. The nomogram containing the above five predictors can accurately predict the risk of residual LBP after surgery.

## 3. Lumbar interbody fusion

Lumbar fusion is currently mainly used for the treatment of LDDs with spinal instability. Anterior, posterior, and side approaches have been developed. The intervertebral disc is removed and the cage is inserted. Internal fixation may be performed according to the patient's condition.

### 3.1. MIS-TLIF

MIS-TLIF is performed using the Wiltse approach, which barely damages the paraspinal muscles, with less blood loss and fast recovery.

Han et al. used finite element analysis of biomechanical studies to investigate the optimal number and position of cages in MIS-TLIF. The authors drew the conclusion that single long-cage transversal implantation is a promising standard implantation method, and double short-cage implantation is recommended for patients with severe osteoporosis in MIS-TLIF.

Zhang et al. evaluated the efficacy, safety, feasibility, and biomechanical stability of contralateral bridge fixation of freehand minimally invasive pedicle screws (freehand MIPS) combined with unilateral MIS-TLIF (smile-face surgery) and open TLIF for the treatment of multi-segmental LDDs. The smile-face surgery group showed a shorter operation time, shorter incision, lower blood loss, and a shorter hospital stay than the open TLIF group (*P* < 0.05). The back VAS score in the smile-face surgery group was significantly lower than that in the open TLIF group immediately and 3 months after surgery, and no significant difference was observed 1, 2, and 5 years after surgery. At the 5-year follow-up, grade I or II fusion was achieved in 99.00% (100/101) of segments in the smile-face surgery group and in 97.67% (84/86) of segments in the open TLIF group, according to the Bridwell system. The complication rate of open TLIF was higher than that of smile-face surgery (24.32% vs. 0%, *P* < 0.01). The results indicated that it is a good choice of treatment for multi-segmental LDDs. Both methods can achieve good biomechanical stability.

### 3.2. Oblique lumbar interbody fusion (OLIF)

OLIF uses the natural retroperitoneal space to reach the intervertebral disc for surgery, avoiding the paravertebral muscles and large blood vessels, and has the advantages of less trauma, quick recovery of postoperative back pain, and a lower complication rate.

Wang et al. investigated the efficacy of stand-alone OLIF vs. combined with percutaneous pedicle screw fixation (PPSF) for the treatment of discogenic lower back pain (DBP). The mean surgery duration, blood loss, and muscle damage in the stand-alone OLIF group were significantly better than those in the OLIF + PPSF group (*P* < 0.05), showing that stand-alone OLIF and OLIF + PPSF are both safe and effective methods for the treatment of DBP; there is no significant difference in the long-term clinical and radiological outcomes. Stand-alone OLIF has the advantages of surgery duration, blood loss, muscle damage, and early clinical effect. This study provides a basis for the clinical application of standard DBP treatment with OLIF.

Li et al. compared differences in the correction effect for the lumbosacral lordosis effect and clinical outcomes between OLIF with/without posterior pedicle screw fixation (PSF) and MIS-TLIF through a retrospective cohort study. The average operation time and intraoperative bleeding were significantly lower in the OLIF group than in the MIS-TLIF group (163 ± 68 vs. 233 ± 79 min, 116 ± 148 vs. 434 ± 201 ml, *P* < 0.001). There was no statistically significant difference between the OLIF group and the MIS-TLIF group in terms of VAS and ODI score improvements, fusion rate, complication, and LL and FSL correction. The results showed that OLIF and MIS-TLIF are both safe and effective procedures, capable of restoring lumbosacral lordosis and disc height partly. Combined with PSF, OLIF can achieve a better correction of lumbosacral lordosis than MIS-TLIF.

Pan et al. used a novel modified OLIF technique (anteroinferior psoas approach, AIPA) or the mini-open, lateral transpsoas approach (LTPA) for anterior decompression reconstruction to treat 68 patients with L1–L4 burst fractures. One-stage monosegmental posterior/anterior surgery was performed. The authors concluded that anterior decompression reconstruction via mini-open AIPA was a safe and less invasive approach, with fewer approach-related complications than LTPA.

Han et al. summarized a technical note on OLIF as a salvage surgery and the preliminary outcomes of a series of cases. The authors retrospectively reviewed patients with leg or back pain induced by pseudarthrosis or adjacent segment disease after PLIF/TLIF. These patients underwent salvage OLIF surgeries. The study showed that OLIF provides a safe and effective salvage strategy for patients with failed posterior intervertebral fusion surgery. Patients effectively achieved recovered intervertebral and foraminal height, with no additional posterior direct decompression.

### 3.3. Percutaneous endoscopic lumbar interbody fusion (PE-LIF)

With the development of spinal endoscopy, some fusion surgeries began to be performed under endoscopy, such as percutaneous endoscopic posterior lumbar interbody fusion (PE-PLIF) or percutaneous endoscopic transforaminal lumbar interbody fusion (PE-TLIF), which has the advantages of more refined operation and less trauma.

Wang et al. investigated the effectiveness and feasibility of biportal endoscopic decompression, debridement, and interbody fusion, combined with percutaneous screw fixation for lumbar brucellosis spondylitis (LBS). Bony fusion was obtained in all patients at the last follow-up, including 12 cases with grade I and 1 case with grade II, with a fusion rate of 92.31%. The results showed that biportal endoscopic decompression, debridement, and interbody fusion, combined with percutaneous screw fixation is an effective, safe, and viable surgical procedure that should be considered for the treatment of LBS.

Feng et al. investigated the clinical efficacy and technical points of percutaneous coaxial large-channel endoscopic lumbar interbody fusion (PCLE-LIF). The authors performed PCLE-LIF to treat 62 cases of single-segment degenerative lumbar spinal stenosis. The interbody fusion rate was 93.5% 1 year after operation, showing that PCLE-LIF for the treatment of degenerative lumbar spinal stenosis has good short-term efficacy and high safety and is worthy of popularization.

## 4. Mini-open or channel assisted surgery

Cui et al. reported a case of surgical treatment for Brucella spondylitis (BS). A negative pressure wound therapy (NPWT) device was introduced into the intervertebral space after the removal of the lesion through the extreme lateral approach. Three weeks after the first operation, fusion was performed using lateral plate fixation to the iliac bone through the original incision to restore the stability of the spine.

Liu et al. explored the clinical effect and operating skills of channel-assisted cervical key hole technology combined with ultrasonic bone osteotome (CKH-UBO) for the treatment of single segment cervical spondylotic radiculopathy (CSR). The conclusion was that channel-assisted CKH-UBO for single segment CSR has the advantages of short operation time, reliable clinical effect, high safety, and low complication rate, which is worthy of clinical promotion.

## 5. Navigation-assisted spine surgery

Huang et al. performed a retrospective study to compare the results between navigation and non-navigation groups and to explore the benefits of unilateral biportal endoscopic lumbar interbody fusion (BE-LIF) assisted by intraoperative O-arm total navigation. The results showed that, compared with the non-navigation approach, the O-arm total navigation–assisted BE-LIF technology not only has similar clinical results, but also can provide accurate intraoperative guidance and help spinal surgeons achieve accurate decompression. Furthermore, it can reduce radiation exposure to surgeons and operation time, improving the efficiency and safety of surgery.

Shi et al. provided detailed information about the improvement of three-dimensional (3D)–printed navigation templates for modified cortical bone trajectory (CBT) screw placement in the lumbar spine and evaluated the safety and accuracy. The authors designed a safe insertion angle, screw diameter, and other indexes through 3D reconstruction and reverse engineering techniques and utilized a 3D printing technique to verify the anatomical vertebra and navigation template. The authors proved that this technique makes it easier and safer for spine surgeons without any experience to place screws using a navigation template. In clinical practice, their 3D printed navigation template and special tools can further improve the accuracy and safety of modified CBT screw placement.

Shi et al. addressed previous and current applications of augmented reality (AR) in MISS, the limitations of today's technology, and future areas of innovation in a literature review. AR systems have been implemented for treatments related to spinal surgeries in recent years, and AR may be an alternative to current approaches such as traditional navigation, robotically assisted navigation (RAN), fluoroscopic guidance, and freehand. Since AR is capable of projecting patient anatomy directly on the surgical field, it can eliminate concerns regarding surgeon attention shift from the surgical field to navigated remote screens, line -of- sight interruption, and cumulative radiation exposure, as demand for MISS increases. The authors drew the conclusion that AR is a novel technology that can improve spinal surgery and will likely have a great impact on future technology.

## 6. Hybrid minimally invasive surgical techniques

Zhou et al. used PTES combined with mini-incision OLIF and anterolateral screw rod fixation for surgical treatment of lumbar spondylolisthesis, and evaluated the feasibility, efficacy, and safety of this method compared with MIS-TLIF. The results showed that PTES combined with mini-incision OLIF and anterolateral screw rod fixation has some advantages over MIS-TLIF, including smaller aggression, lower blood loss, lower operative duration under general anesthesia, quicker postoperative back pain relief, better restoration of sagittal lumbar parameter, and better fusion. For both methods, the long-term clinical efficacy and complication rate are comparable. PTES combined with mini-incision OLIF and anterolateral screw rod fixation is a good choice of minimally invasive surgery for lumbar spondylolisthesis, which barely destroys the paraspinal muscles and bone structures.

## 7. Others

Wu et al. retrospectively analyzed 22 patients who underwent modified double door laminoplasty based on Shirashi's method. During the procedure, laminar grooves were made on both sides through the segmental muscle space. The spinous process was split while retaining the muscle attachment point. After opening the door, the central gap was fixed with a self-developed titanium mini plate. Patients who underwent this surgical approach had preserved posterior muscles and this prevented obvious axial symptoms and improved their quality of life.

These 30 accepted manuscripts in this Research Topic have generated an updated concept of MISS, described novel techniques, and optimized MISS procedures for various spinal diseases, which may further encourage the development of additional innovations in the field.
